# Insights into metabolic and pharmacological profiling of *Aspergillus ficuum* through bioinformatics and experimental techniques

**DOI:** 10.1186/s12866-022-02693-w

**Published:** 2022-12-09

**Authors:** Zafar Ali Shah, Khalid Khan, Haroon Ur Rashid, Tanzeel Shah, Mariusz Jaremko, Zafar Iqbal

**Affiliations:** 1 Department of Chemistry, Islamia College, Peshawar, KP Pakistan; 2 Department of Agricultural Chemistry & Biochemistry, University of Agriculture, Peshawar, KP Pakistan; 3 Department of Chemistry, Federal University of Santa Catarina, Florianopolis, SC Brazil; 4 Institute of Basic Medical Sciences, Khyber Medical University, Peshawar, KP Pakistan; 5 Biological and Environmental Science and Engineering Division, King Abdullah University of Science and Technology, Thuwal, Makkah Province 23955-6900 Saudi Arabia

**Keywords:** Metabolic Profiling, Pharmacological screening, Molecular docking, *Aspergillus ficuum*

## Abstract

**Background:**

Recently, numerous novel bioactive fungal metabolites have been identified that possess broad therapeutic activities including anti-inflammatory, antibiotic, antioxidant, and antitumor. The fungal mycochemicals as well as extracts have increased the interest of the scientific community in drug discovery research through a combination approach such as, molecular metabolic, pharmacological and computational techniques. Therefore, the natural fungus *Aspergillus ficuum* (*A. ficuum*) (FCBP-DNA-1266) was selected for metabolic and pharmacological profiling in this study.

**Results:**

The metabolic profile of *A. ficuum* was explored for the first time and revealed the presence of bioactive compounds such as choline sulfate, noruron, hydroxyvittatine, aurasperone D, cetrimonium, kurilensoside, heneicosane, nonadecane and eicosane. Similarly, a pharmacological screen of *A. ficuum* was performed for the first time in *in vivo* and *in vitro* models. Interestingly, both the ethyl acetate and n-hexane fractions of *A. ficuum* were found to be more active against *Bacillus subtilis* among five tested bacteria with their zone of inhibition (ZOI) values of 21.00 mm ±1.00 and 23.00 mm ±1.00, at a concentration of 150 μgmL^-1^ respectively. Similarly, a significant decrease (*P*<0.001) and (*P*<0.01) in paw edema was observed in *A. ficuum*-treated animals at doses of 50 and 150 mgkg^-1^, respectively, reflecting its potent anti-inflammatory effect. Furthermore, the docking results supported the antibacterial and anti-inflammatory effects of *A. ficuum*. In addition, the crude extract demonstrated no acute toxicity and the highest percent radical scavenging was recorded for both n-hexane and ethyl acetate extracts.

**Conclusion:**

The metabolic profile of *A. ficuum* indicated the presence of biological relevant compounds. *A. ficuum* extract exhibited potent antibacterial and anti-inflammatory effects supported by docking results. Furthermore, *A. ficuum* extract demonstrated the highest percentage of radical scavenging activity along with no acute toxicity.

**Supplementary Information:**

The online version contains supplementary material available at 10.1186/s12866-022-02693-w.

## Introduction

Human life is under threat worldwide as mortality increases in developing countries. The spread and development of infectious diseases and drug-resistant pathogens has stimulated the search for new pharmaceutical agents [1]. The development of novel bioactive drugs is not only required against microbial resistance but also very important to overcome degenerative diseases. As a degenerative disease, problems are caused by the reactive oxygen species (ROS) and are detrimental to various biological processes involved in human health [2]. A natural product, schisandrin A, which belongs to the lignan class, significantly attenuated DNA damage and H_2_O_2_-induced cytotoxicity by blocking ROS accumulation [3]. Similarly, triterpenoids are diverse molecules and are considered ROS modulators [4]. Since the discovery of penicillin from *Penicillium,* the exploration of antibiotics and other drugs from microorganisms like fingolimod, cyclosporine, lovastatin, caspofungin, etc. has become popular in the pharmaceutical industry. Fungi have been reported for the production and development of different classes of bioactive metabolites [5].

Recently, numerous new bioactive fungal metabolites have been discovered that have wide-ranging therapeutic properties, such as: anti-inflammatory, antibacterial, antioxidant and anti-cancer properties. The scientific community's interest in drug discovery research has grown as a result of the use of fungal mycochemical chemicals and extracts for various purposes [6].

The genus *Aspergillus* consists of several fungi known in the pharmaceutical industry for their important therapeutic role. They have diverse chemical applications as shown by genome sequencing. The fungal secondary metabolites are controlled by the genes present in the biosynthetic gene clusters (BGCs), which are chromosomal architectures encoding the required synthetases and/or synthases in the fungal genome [7]. In recent years, more than 315 bioactive metabolites based on the unique chemical structure of fungal species of the genus *Aspergillus* have been documented [8]. Therefore, *A. ficuum* is selected for metabolic and pharmacological profiling for the first time in this study. *A. ficuum* is known as black *Aspergilli* which belongs to the Niger group of fungi. Since 1923, the Niger group has been used explicitly for commercial purposes in the production of cosmetics, pharmaceutical agents, and citric acid. A large number of secondary metabolites and mycotoxins are reported in *niger* clad including ochratoxins, fumonisins, naphtha-pyrones, bicoumarins, malformins, asperazines, and alkaloids [9, 10]. Recently, a protein profile study of *Aspergillus niger* was performed by using advanced technique of nLC-qTOF mass spectrometry; a total number of 108 protein molecules were reported from *Aspergillus niger* [11].

Our work is related to uncover the untargeted metabolites of less-studied *A. ficuum* that are biosynthesized in liquid culture through Liquid chromatography quadruple time-of-flight mass spectrometry (LC-QToF-MS) and Gas chromatography-mass spectrometry **(**GC-MS) analysis. Further, the pharmacological potential such as, anti-inflammatory, acute oral toxicity, antibacterial and free radical scavenging potential of both ethyl acetate and the n-hexane fractions of *A. ficuum* were also investigated applying *in vivo* and *in vitro* models, respectively. In addition, the proposed mechanism through which tentatively identified mycocompounds interact with DNA-polymerase enzyme of the *Bacillus subtilis* and the inflammation supporting enzyme cyclooxygenase-2 was also investigated by molecular docking analysis.

## Materials and Methods

### Fungal strains and animals

The fungal strain *Aspergillus ficuum* (FCBP-DNA-1266) was purchased from the First Culture Bank of Pakistan, University of Punjab, Lahore [12]. Mice of both sexes (BALB/c) 25-35 g were purchased from the National Institute of Health (NIH), Islamabad, Pakistan. They were bread and maintained on a light/dark cycle spanning 12 h/12 ​​h at 22 °C, with water and food provided via ad libitum throughout the study. The Animal Scientific Procedures Act, NIH (the UK, 1986) guidelines were followed throughout the experiments for the care and laboratory use of the animals [13]. The study protocols for *in vivo* studies were approved from the Ethical Committee FAHV&S, under the number 7196/LM/UoA, The University of Agriculture Peshawar, Pakistan.

### Extraction and fractionations

Spores (10^5^ conidia/mL) from the actively growing culture of *A. ficuum* were transferred to sterilized 30 L of Potato Dextrose Broth (PDB) in multiple 500 mL Erlenmeyer flasks. The flasks were held static at 28 °C ± 2 for 21 days. The mycelium obtained from each flask was dried and crushed into powder form. The powdered mycelia were extracted with ethyl acetate (EtOAc) (3×300 mL) followed by fractionation with n-hexane (3×300 mL) (solvent-solvent extraction). The fractionation was performed using a separatory funnel. The ethyl acetate fraction, which is denser than n-hexane, was removed first, followed by the n-hexane fraction. Both the fractions were condensed under reduced pressure using a rotary evaporator (Buchi, Germany, Model R-300). Both the dried fractions ethyl acetate (2.3 g) and (1.7 g) were stored in the refrigerator at 4 °C for further analysis [14].

### LC-QToF-MS analysis

Untargeted metabolic profiling of *A. ficuum* was performed by liquid chromatography quadruple time-of-flight mass spectrometry (LC-QToF-MS). A crude extract of 0.5 mg was dissolved in 1 mL of methanol and loaded to the column. Before analysis, the crude extract solution was filtered through a 0.45 micron filter. The assembly consisted of a diode array detector, quaternary pump, autosampler cooled chambers (4 °C), degasser, and controlled temperature column (25 °C) coupled to quadruple time-of-flight (QToF) mass spectrometer (MS) (Agilent 6530) with steam ionization source of a dual jet (Agilent Technologies, Australia). The positive ionization mode was acquired with the help of the full scan mass spectrum. C18 (Poroshell column) with the guard column (Agilent Technologies) was used for chromatographic separation. Subsequent separation was done on column (Kinetex HILIC) (Phenomenex, USA). The flow rate of the mobile phase was 0.3 mL min^-1^. The columns were equilibrated for 40 min before analysis. The gradients of solvent A (LCMS grade) [water (Milli-Q, Germany) + 0.1% formic acid (Sigma-Aldrich)] and solvent B [acetonitrile (95% HPLC grade, Thailand) + 0.1% formic acid (Sigma-Aldrich)] was used for separating the flow rate maintained at 0.3 mL min^-1^. The solvent gradient was as follows: 5% B for 0.5 min, increasing to 100% B over the next 16.5 min, then holding at 100% B for 23 min, and returning to 5% B from 23.1 min to 29 min. DAD monitored the absorbance in the wavelengths ranging from 210-635 nm. The QToF-MS was standardized at psi 35. Fragmentor and capillary voltage were kept at 135 V and 3500 V, respectively. The injection volume for crude ethyl acetate extract of *A. ficuum* was 10 μL. Three collision energies were used to record MS/MS spectra by using Auto MS/MS mode. UHPLC-QToF-MS was used for compound quantification. The algorithm set for matching secondary metabolites with Personal Compound Database Library (PCDL) (Agilent Technologies, CA, USA) was entities having similar accurate mass and retention time [15].

### Gas Chromatography-Mass Spectrometry (GC-MS) Analysis

GC-MS analysis of crude *A. ficuum* extracts was performed on an Agilent 7890A/5975C. A stock solution of crude extract 10 μgmL^-1^ was prepared. For analysis, 50 μL of the solution was filtered and loaded to the column using injection. The capillary column was operated in EI mode. An MS column HP-5 (30 m×250 m×0.25 m) was used as the stationary phase. A helium carrier gas with a flow rate of 1.2 mLmin^-1^ was used as a mobile phase. The peak area expresses the concentration of the chemical components in the extract, while the retention indices help identify the compounds. A different runtime was provided. Mass spectra (EI) were examined at 70 eV keeping the scan range between 35-650 amu. The obtained mass spectrum was coordinated with NIST 08. L mass spectrum libraries [16]. The result was thoroughly inspected and the impurity peaks were removed. Also, the repetition of compounds or match names at different retention times was removed and the obtained data were simplified.

### Antibacterial assay

Pathogenic strains, including gram-negative bacteria *Escherichia coli*, *Xanthomonas campestris,* and the gram-positive bacteria *Clavibacter michiganensis, Bacillus subtilis, Staphylococcus aureus* were investigated for antibacterial activity. Fresh bacterial cultures were grown in nutrient broth at 37 °C for 24 h. Sterilized disks were soaked in 60 μL fungal extract of ethyl acetate and n-hexane having a concentration of 150 μgmL^-1^. Streptomycin was considered a positive control and dimethyl sulfoxide (DMSO) as a negative control. The antimicrobial assay was assessed by measuring the zone of inhibition in millimeters. The extent of inhibition compared to the control was calculated as follows [17].

$$\begin{array}{c}\mathrm{Percentageofinhibition}=\mathrm{CI}-\mathrm{EI}/\mathrm{CI}\ast100\\\mathrm{Where}\;\mathrm{CI}\;=\;\mathrm{control}\;\mathrm{inhibition}\;\mathrm{and}\;\mathrm{EI}\;=\;\mathrm{extract}\;\mathrm{inhibition}.\end{array}$$

### DPPH radical scavenging assay

Percent radical scavenging activity was evaluated by DPPH (2, 2`-Diphenyl-1-picrylhydrazyl) protocol [18]. Different concentrations of fungal extracts from *A. ficuum* of 25, 50, 75, and 100 g mL^-1^ were prepared in DMSO. A DPPH solution (0.1 mM) with a volume of 2.96 mL was prepared in methanol and was added to both fungal extracts. The solution was kept in the dark for half an hour and the absorbance maxima were noted at 517 nm using a spectrophotometer (6800 UV-VIS spectrophotometer). The percentage (%) of DPPH radical scavenging was measured using the following formula:

$$\%\mathrm{RSA}=100-\lbrack(\mathrm{As}/\mathrm{Ac})\times100\rbrack$$

Where As= sample absorbance

Ac= negative control absorbance

### Anti-inflammatory assay

The carrageenan-induced paw edema test was used to evaluate the anti-inflammatory activity of ethyl acetate extract of *A. ficuum*. The animals were divided into four groups such as, vehicle, vehicle + carrageenan, aspirin + carrageenan, and *A. ficuum* + carrageenan. The ethyl acetate extract of *A. ficuum* was orally administered in two different doses of 50 mgkg^-1^ and 150 mgkg^-1^ together with aspirin (control) at a dose concentration of 150 mgkg^-1^ to the respective groups of mice. The doses of *A. ficuum* tested for anti-inflammatory activity were estimated for consideration from the reported studies [19, 20]. These concentrations were optimized for important variables and assessed to identify the stated values. After one hour, 5 mL of a 1% carrageenan solution was administered to the left hind paw in the subplantar area. The anti-inflammatory effect was assessed using calipers by measuring the paw thickness of the respective animals. This process of measuring paw thickness was repeated every hour for a total of 5 hours of study. The percentage anti-inflammatory effect was evaluated by the following Equation [21].

$$\begin{array}{c}\mathrm{Anti}-\mathrm{inflammatory}\;\mathrm{effect}\;(\%)\;=\;\lbrack(\mathrm{Ct}\;-\;\mathrm C0)\;\mathrm{control}\;-\;(\mathrm{Ct}\;-\;\mathrm C0)\;\mathrm{treated})\rbrack\;/\;(\mathrm{Ct}\;-\;\mathrm C0)\;\mathrm{control}\;\times\;100\\ \mathrm{Ct}\;=\;\mathrm{thickness}\;\mathrm{after}\;1-5\;\mathrm h\;\;\;\;\;\;\mathrm C0\;=\;\mathrm{baseline}\;\mathrm{paw}\;\mathrm{thickness}.\end{array}$$

### Acute oral toxicity

Acute oral toxicity of *A. ficuum* extract was performed on mice. Random groups were formed, each consisting of six animals (25-30 g). They were subjected to 12-hour fasting. Different doses of the extract i.e. 10 mLkg^-1^, 15 mLkg^-1,^ and 20 mLkg^-1^ dissolved in normal saline were administered orally via a gavage. The control group received normal saline at a concentration of 10 mLkg^-1^. All animals were given free access to the water. Feeding and observation were carried out in two phases, viz. initially six hours and then 72 hours for the presence of acute toxicity manifestations. Daily visual observations of growth, general behavior and physical activity, mortality, morbidity, and general health were identified and documented [21]. From the published research [22, 23], estimates were made for the *A. ficuum* doses tested for acute oral toxicity. These concentrations were evaluated to determine the reported values and optimized for significant variables.

### Molecular docking analysis

Mycocompounds tentatively identified through LC-QToF-MS investigation were selected for docking study against DNA-polymerase (PDB ID: 4TR6) enzyme of *Bacillus subtilis* and the inflammation supporting enzyme cyclooxygenase-2 (COX-2) (PDB ID: 5JVZ) [24, 25]. High-resolution X-rays crystal structures of the selected enzymes were imported from Protein Data Bank (HTTP:// www.rcsb.org/pdb). Further protein molecules were prepared for docking analysis by removing water molecules, adding missing hydrogen atoms, assigning of correct hybridization state to each atom in each residue, and correcting charges applying the preparation program embedded in Molecular Operating Environment (MOE) software. Active sites inside the selected proteins were identified through the active site finder tool of MOE software. Finally, tentatively identified compounds were docked inside the active binding sites of the selected pathogenic proteins employing the docking program of MOE software. Thirty conformations were generated for each compound through selected torsion angles for all the rotatable bonds of the compound. London dock scoring function implanted in MOE was applied to find out the binding energy for each protein-ligand complex system [26, 27].

## Results & Discussion

### Metabolites profiling through LC-QToF MS analysis

**Fig. 1:**
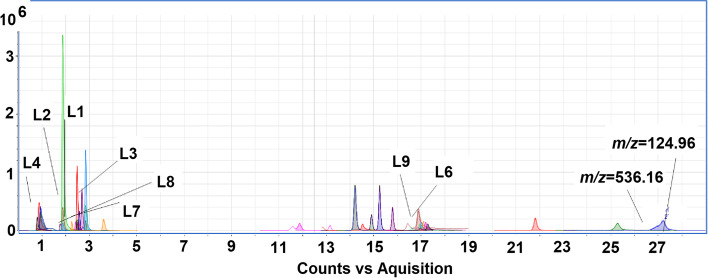
LC- QToF-MS (ESI^+^) chromatogram of *A. ficuum*

In this study, an untargeted metabolic profiling of *A. ficuum* was investigated for the first time, showing satisfactory data quality with high specificity and sensitivity (Table S1). In the positive ion mode of the *A. ficuum* extract, 35 distinct chromatographic peaks were observed (Fig. 1). Twenty-one compounds were identified using METLIN and Personal Compound Database Library (PCDL), while the remaining fourteen peaks remained unidentified. In this research, we have elaborated the 9 major secondary metabolites identified by LC-QToF-MS analysis on the basis of 99% match score of molecular mass with the libraries (Table 1).

**Table 1:** LC-QToF-MS analysis of ethyl acetate extract of *Aspergillus ficuum*

Compounds^a^	Name	Formula	RT	*m/z*
L1	11-Hydroxyvittatine	C_16_ H_17_ N O_4_	2.485	288.1232
L2	Noruron	C_13_ H_22_ N_2_ O	1.882	223.1806
L3	Aurasperone D	C_31_ H_24_ O_10_	3.601	557.1446
L4	Choline sulfate	C_5_ H_14_ N O_4_ S	0.947	184.0639
L5	4-[[5-[[(cyclopentyloxy)carbonyl] amino]-1-methyl-1H-indol-3-yl] methyl]-3-methoxy benzoic acid	C_24_ H_26_ N_2_ O_5_	2.842	423.1922
L6	Kurilensoside F	C_33_ H_58_ O_11_	17.108	648.432
L7	17-phenyl-trinor-PGE2	C_23_ H_30_ O_5_	2.487	409.1971
L8	4-Benzyloxy-2'-hydroxy-3',4',5',6'-tetramethoxychalcone	C_26_ H_26_ O_7_	2.839	901.3418
L9	Cetrimonium	C_19_ H_42_ N	16.888	284.3316

^a^Compounds=Ligands

It was also observed that some metabolites were previously only reported as being of plant origin. Most species of fungi have a mutual relationship with plants. According to the study, plant enzymes have the potential to alter fungal gene expression in a mutual relationship [28]. This fungal-host relationship is responsible for the production of different compositions of metabolites [29]. The compound 11-hydroxyvittatin is a phyto-alkaloid and has already been described from many plant species [30]. Benzyloxy-2'-hydroxy-3',4',5',6' tetramethoxychalcone belongs to the class of flavonoids. Many flavonoids are reported from endophytic fungi. Flavonoids from endophytic fungi are known to play an important role in the production of reactive oxygen species (ROS) and reactive nitrogen species (RNS) that help plants protect themselves from microbial attacks [31].

Interestingly, to the best of our knowledge, some metabolites such as, Kurilensoside F, beclomethasone, 4-[[5-[[(cyclopentyloxy)carbonyl]amino]-1-methyl-1H-indol-3-yl]methyl]-3-methoxy-benzoic acid (zafirlukast) and 4,4',6-trimethylangelicin have not previously been reported from microbial or plant origin and are being reported from this species for the first time. This change might be due to geographic origin, ecological, climatic, and nutritional needs [32]. Kurilensoside F is a prominent important biological compound which was first documented from starfish [33]. Two other important anti-asthmatic compounds were beclomethasone and 4-[[5-[[(cyclopentyloxy)carbonyl]amino]-1-methyl-1H-indol-3-yl]methyl]-3-methoxy-benzoic acid (zafirlukast) used as medicines against lung diseases [34, 35]. Additionally, 4,4',6-trimethylangelicin is a furocoumarin that appears to be a promising drug for photochemotherapy of psoriasis [36].

Some peaks with *m/z* values of 239.2118, 129.1388, 665.2874, 457.1769, 571.1605, 736.4843, 692.4578, 604.4055, 560.3798, 536.1659 and 124 .9642 were not identified by the METLIN and PTDC. The data obtained showed that some chemical constituents of *A. ficuum* are already been reported from Niger clad. The Niger clade consists of different species such as, *A. uvarum*, *A. japonicus*, *A. homomorphus*, *A. aculeatus*, *A. aculeatinus* and *A. ellipticus* [9]. These compounds include aurasperone D and deoxysappanone B from the naphtho-ɣ-pyrones class and are best known for their potent cytotoxic potential [37]. Given the need to discover new medicines, synthetic and plant-derived medicines are not enough to meet the specific needs of today's world. Therefore, *A. ficuum* could be a promising candidate to take on its role in the pharmaceutical industry for a future drug discovery program.

### GC-MS analysis

**Fig. 2:**
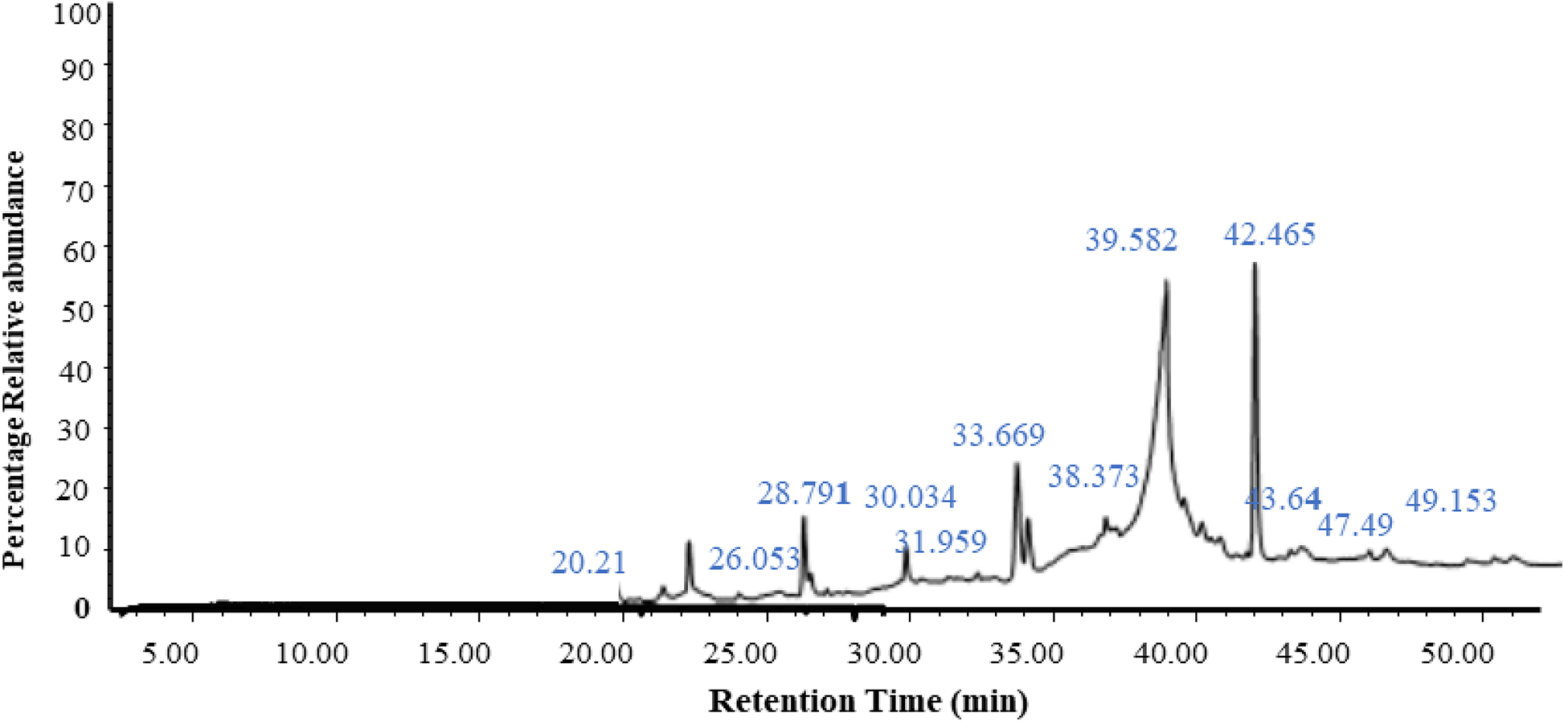
GCMS Chromatogram of *A. ficuum*

A well-defined total ion chromatogram (TIC) of *A. ficuum* ethyl acetate extract represents compounds of different classes (Fig. 2) such as, alkanes, alkenes, alcohols, amides, aromatics, and organosulphur (Table S2).

Bioactive compounds (Table 2) potentially having broad therapeutic and other beneficial properties present in ethyl acetate extract were tentatively identified by GC-MS analysis. Compounds such as, heneicosane, nonadecane, eicosane and furan derivatives known for their pheromonic, antimicrobial activities and fumigating properties have been identified [38, 39].

**Table 2:** GC-MS analysis of ethyl acetate extract of *Aspergillus ficuum*

Retention time	Number of compounds	Match name	M.wt (amu)
21.675	1	1-Tetradecene	196.219
26.053	1	1-Octadecene	252.282
28.791	2	E-15-Heptadecenal	252.245
		Hexadecanoic acid, methyl ester	270.256
30.034	1	Cycloeicosane	280.313
31.959	7	9,12-Octadecadienoic acid (Z, Z)-, methyl ester	294.256
33.669	11	1-Docosene	308.344
		1-Eicosene	280.313
		1-Pentadecanethiol	244.222
		1-Nonadecene	244.222
		n-Nonadecanol-1	284.308
		Tridecanoic acid, methyl ester	228.209
		9,17-Octadecadienal, (Z)	264.245
		Heptadecanoic acid, 16-methyl-, methyl ester	298.287
		1-Heneicosanol	312.339
		1-Hexadecanol	242.261
		11-Tricosene	322.36
39.582	2	1,2-Benzenedicarboxylic acid, mono(2-ethylhexyl) ester	278.152
38.373	1	7-Pentadecyne	208.219

A study conducted on 9,12-Octadecadienoic acid found that it inhibited glucose production in H4IIE cells at 25 μM, which is a good sign for antidiabetic studies [40]. Insecticidal activity has been reported from the essential oil containing heneicosane, eicosane and other fatty acid methyl ester [41]. Previously, various compounds were tentatively identified by GC-MS analysis in numerous species of the *Aspergillus* genus such as, *A. clavatonanicus* and *A. niger* [42, 43]. However, this study also reported 26 bioactive molecules in *A. ficuum*-a specie belonging to the same genus *Aspergillus*.

### Antibacterial activity

**Fig. 3:**
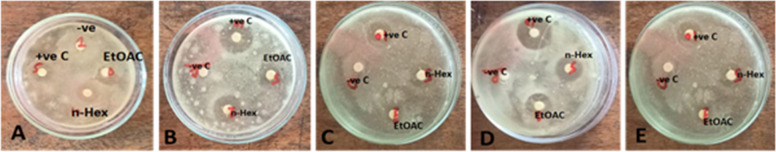
Antibacterial activities of EtOAc and n-Hexane extract of *Aspergillus ficuum*, **A** *Escherichia coli,* **B** *Staphylococcus aureus*, **C** *Clavibacter michiganensis*, **D**=*Bacillus subtilis*, **E** *Xanthomonas campestris*

Bioactive mycocompounds were tentatively identified in *A. ficuum* extract through LC-QToF-MS and GC-MS analysis. Therefore, a preliminary antibacterial screening was performed on crude n-hexane and ethyl acetate fractions of *A. ficuum* against five standard pathogenic bacteria (Fig. 3). Zones of inhibition values were obtained ranging from 16-23 mm and 13-21 mm for n-hexane and ethyl acetate fractions, respectively (Table 3**)**. Both n-hexane and ethyl acetate fractions of *A. ficuum* inhibited the growth of all pathogens; they proved to be more effective against *Bacillus subtilis* with their zone of inhibitions (ZOI) of 21.00 ±1.00 and 23.00 ±1.00, respectively. These results are attributed to the antimicrobial potency of mycocompounds present in both fractions of *A. ficuum*. The standard streptomycin was found more active against *Clavibacter michiganensis* with a zone of inhibition of 29.25 mm. As diverse antimicrobial activity has previously been documented from fatty acid methyl ester, the existence of 9,12-Octadecadienoic acid and 9,12-octadecadienoic acid can be linked to the powerful antibacterial impact of n-hexane [44]. The genus *Aspergillus* is an active biosynthesizer of essential pharmacological and industrial products [45]. Many bioactive compounds such as, aurasperone H, butenolide, aspergivones, asperchondols have been reported from *Aspergillus* [46, 47]. The ethyl acetate extract of *aspergillus niger* was also reported to be active against standard bacterial pathogens [48–50].

**Table 3:** Antibacterial inhibitory activity of fractions of *Aspergillus ficuum*

Sample	Conc	Bacterial strains	n-Hexane	Ethyl acetate	Streptomycin
		Zone of Inhibition (mm)			
*Aspergillus ficuum*	10 μgml^-1^	*Escherichia coli*	16.00 ±1.00	13.00 ± 1.00	28 ± 0.288
		*Staphylococcus aureus*	17.67 ±1.53	17.00 ±2.00	26 ± 0.25
		*Clavibacter michiganensis*	22.67 ±2.52	20.67 ±1.15	29.25 ± 1.06
		*Bacillus subtilis*	23.00 ±1.00	21.00 ±1.00	24.66 ± 0.7
		*Xanthomonas campestris*	17.67 ±0.29	12.00 ±1.00	28 ± 0.7

### DPPH Radical scavenging activity

Taking into account the antioxidant potential of *Aspergillus* species, the scavenging potency of both n-hexane and ethyl acetate extracts of *A. ficuum* was evaluated in a concentration-dependent manner using an in vitro model. The results showed that both n-hexane and ethyl acetate fractions of *A. ficuum* have the potential to scavenge the free radicals at all concentrations ranging between 25-100 μg mL^-1^ (Table 4).

**Table 4:** DPPH radical scavenging activity of *Aspergillus ficuum*

Sample	Concentration (μgmL^-1^)	n-Hexane	Ethyl acetate	Ascorbic acid
		Percentage Radical Scavenging Activity		
*Aspergillus ficuum*	25	5.19±0.13	10.82±0.04	45.06±0.03
	50	16.01±0.08	21.64±0.22	63.16±0.08
	75	27.05±0.21	32.46±0.08	77.22±0.12
	100	37.89±0.27	43.29±0.12	88.02±0.07

Both the n-hexane and ethyl acetate fractions had the highest antioxidant potential at 100 μg mL^-1^, i.e., 37.89% and 43.29%, respectively. The radical scavenging nature of both extracts can be credited to the presence of mycocompounds that can donate their electron or proton to stabilize the DPPH radical. In addition, the results also demonstrated that the radical scavenging potential of ethyl acetate extract was higher than that of n-hexane; this is attributed to the presence of a higher concentration of radical scavengers in the ethyl acetate extract. Both the ethyl acetate and n-hexane fractions of *A. ficuum* showed a linear increase with an increase in concentration (Fig. S2). While in the case of standard (ascorbic acid), the highest percent radical scavenging activity was 88.02% at concentration of 100 μg mL^-1^. Microbes are a great source of antioxidant compounds. Secondary metabolites isolated from Aspergillus species are reported to inhibit lipid peroxidation, linoleic acid oxidation for hydrogen peroxide, ABTS, and DPPH radical scavenging activities [51–54].

### Anti-inflammatory activity

**Fig. 4:**
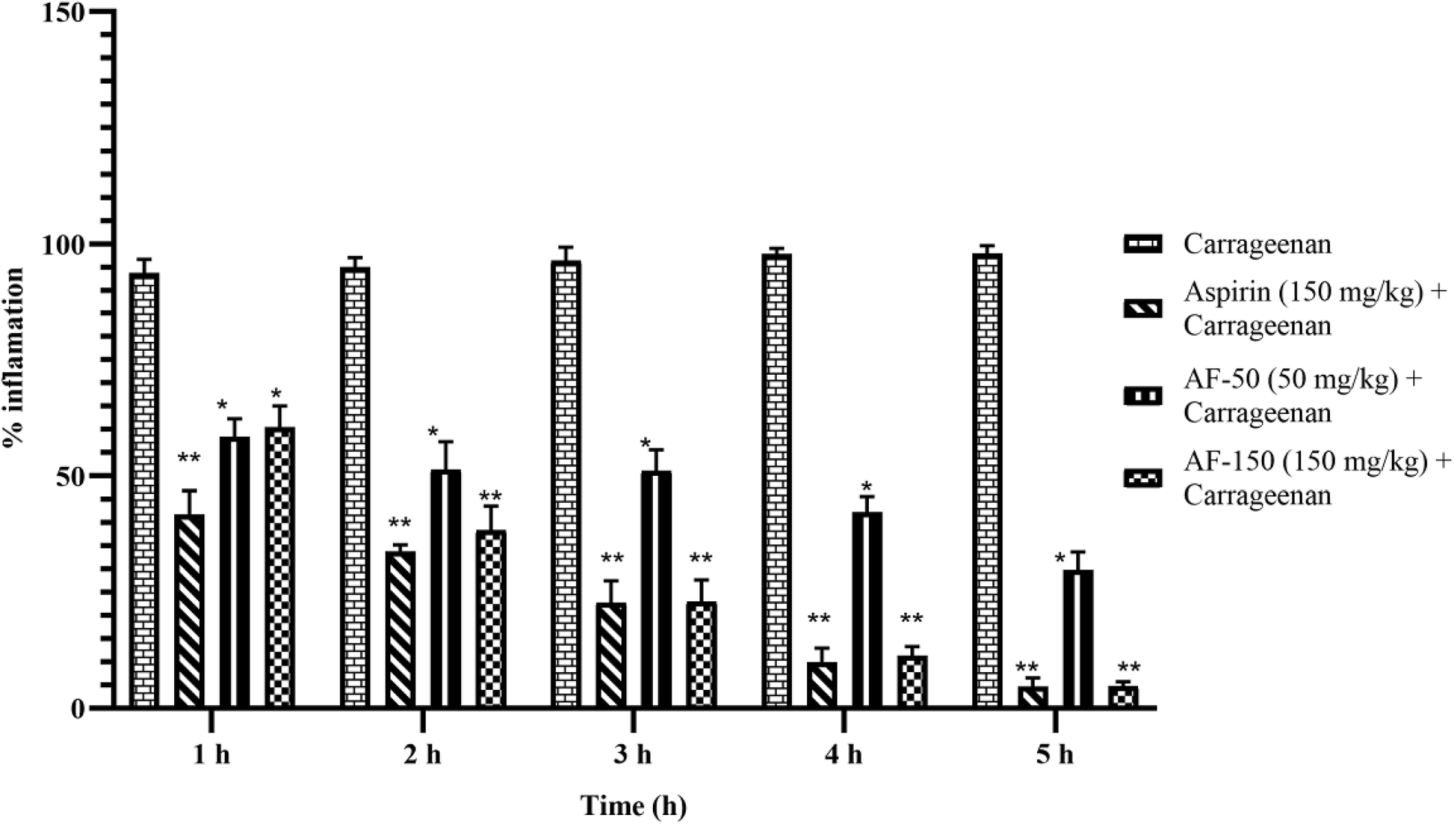
Graphical representation of the anti-inflammatory effect of *A. ficuum* extract

The effects of *A. ficuum* ethyl acetate extract on temporal inflammation in the hind paw of mice were evaluated and induced by intraplantar injection of carrageenan. The results showed a significant inhibitory, dose-dependent response in paw edema compared to vehicle+carrageenan-treated mice or control groups (Fig. 4, S3). A significant decrease (*P* < 0.001) and (*P* < 0.01) in the edematous paw was observed in *A. ficuum*-treated mice at doses of 50 and 150 mg kg^-1^ respectively, during the 5 hour study as compared to carrageenan (Table 5).

**Table 5:** Anti-inflammatory activity of ethyl acetate extract of *Aspergillus ficuum*

Percent Inflammation (%)				
Time	Carrageenan	Aspirin (150 mg kg^-1^) + Carrageenan	A. ficuum (50 mg kg^-1^) + Carrageenan	A. ficuum (150 mg kg^-1^) + Carrageenan
1 h	93.644±3.006	41.71^**^±5.1	58.44^*^±3.821	60.38^*^±4.648
2 h	94.994±2.048	33.81^**^±1.385	51.31^*^±6.028	38.35^**^±5.212
3 h	96.405±2.861	22.76^**^±4.669	51.03^*^±4.563	22.93^**^±4.709
4 h	97.723±1.296	9.94^**^±2.966	42.27^*^±3.282	11.32^**^±1.911
5 h	97.959±1.662	4.68^**^±1.811	29.73^*^±3.905	4.71^**^±0.998

The effects of A. ficuum on paw edema induced by carrageenan. The values are presented as mean ± SEM, the complete randomized design was used and the p-value was calculated using one-way ANOVA. * Represent the *p*-value <0.001 and ** represent *p*-value <0.01 as compared to carrageenan. *n* = 6 mice per group

This clearly shows that *A. ficuum* extract possesses certain chemical constituents that counteract the release of serotonin, kinins, and histamine. In addition, the anti-inflammatory effect of *A. ficuum* was also confirmed by an increase in the percentage inhibition of inflammation. Treatment with aspirin (standard) at a dose of 150 mg kg^-1^ resulted in a significant decrease (*P* < 0.01) in carrageenan time-induced paw edema. Relatively speaking, the aspirin (standard) and *A. ficuum* at a dose level of 150 mgkg^-1^ exhibited a robust anti-inflammatory effect, which is in line with the results of a decrease (*P* < 0.01) in thickness in paw edema and an increase in the percentage inhibition of inflammation (*P* < 0.01), related to the vehicle-treated carrageenan injected animals’ group (Fig. 4).

Several secondary metabolites such as phloroglucinol and glyceollin derived from Aspergillus species have been reported for their anti-inflammatory effect [55, 56]. The polyketides isolated from *A. rugulosa* exhibited anti-inflammatory effects better than the positive control [57]. Similarly, a review of *Aspergillus* genus metabolites revealed that a rich source of anti-inflammatory metabolites are synthesized by *Aspergillus* genus species such as, polyketides, terpenoids, butenolides, and alkaloids [58]. This study will help expand the database of *Aspergillus* anti-inflammatory metabolites and increase knowledge of the mechanism of action of these fungal metabolites.

### Acute toxicity

The acute toxicity of *A. ficuum* extract in mice was studied using an in vivo model (Table 6). Intraperitoneal injection of *Territerum* from *A. terreus* demonstrated its lethality at doses of 17.60 1.91, 9.06 1.07 and 6.28 1.56 mgkg^-1^. Therefore, no mortality was recorded for either the negative control or the mushroom extracts in the 72-hour study at doses of 10, 15 or 20 mlkg^-1^. Further studies at higher doses are needed to confirm the safe use of *A. ficuum* extract in therapeutics.

**Table 6:** Acute toxicity of ethyl acetate extract of *Aspergillus ficuum*

Treatment	Dose (ml kg^-1^)	Replication	Time Interval (h)	No of the mice died
Negative control	10	1	72	0/5
*A. ficuum*	10	1	72	0/5
	15	2	72	0/5
	20	3	72	0/5

The genus *Aspergillus* is known to produce mycotoxins which are responsible for the lethality of fungal species. Filamentous fungi produce a range of mycotoxins; Ochratoxin A is only considered because of its toxicity [59]. The most potent *Aspergillus*-derived mycotoxins include ochratoxins, aflatoxins, sterigmatocystin, patulin, gliotoxin, and fumonisins. Mycotoxins are believed to have cytotoxic, nephrotoxic, teratogenic, carcinogenic and genotoxic properties that can lead to immunocompromised conditions, renal dysfunction and liver cancer [23]. The biological activity of *A. ficuum* without toxic effects indicates that this fungus has the potential to be used as an active pharmaceutical ingredient in future drug discovery.

### Docking studies

**Fig. 5:**
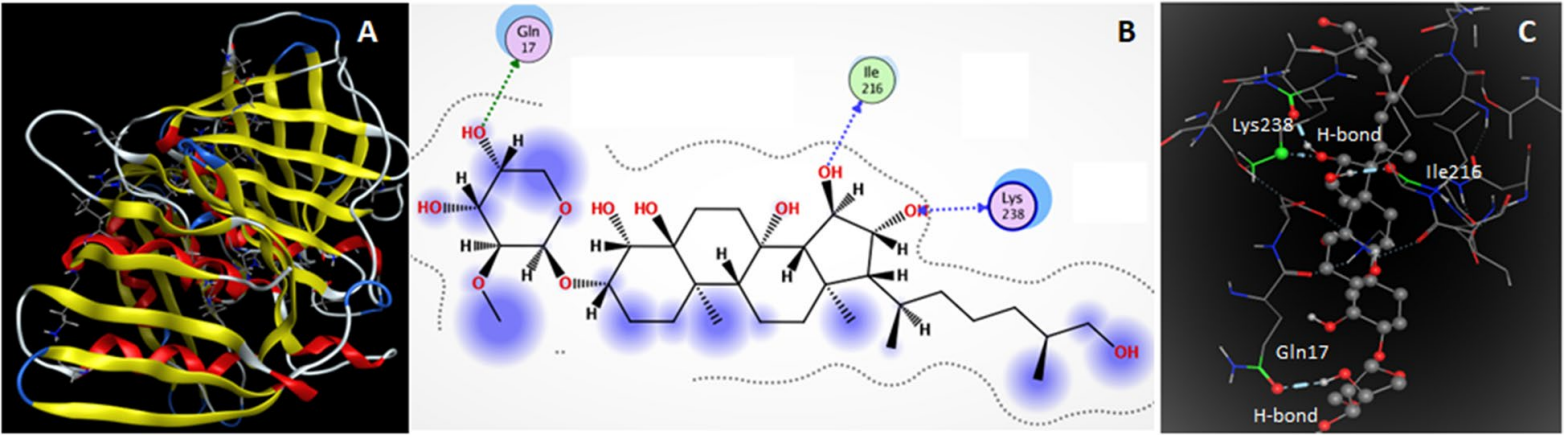
(**A**) 3D structures of 4TR6, (**B**) 2D and (**C**) 3D interactions of L6 with DNA-polymerase enzymes of *Bacillus subtilis* (D stands for dimensional)

For supporting antibacterial and anti-inflammatory activities, tentatively identified mycocompounds and receptor molecules i.e. DNA-polymerase enzyme of *Bacillus subtilis* and inflammation supporting enzyme namely cyclooxygenase-2 (COX-2) were docked. The DNA-polymerase enzyme mainly catalyzes the process of replication of the genome accurately and efficiently; further it is responsible for the maintenance and transmission of genetic information. COX-2 catalyzes the process of conversion of arachidonic acid to thromboxanes and prostaglandins upon the induction of inflammation that might harm the tissues. Docking results (Table 7) reveal that myco-molecules (L1-L9) show strong interactions with both enzymes 4TR6 and 5JVZ. Among all compounds, ligand L6 (kurilenoside F) has the highest binding energy of -9.9476 Kcalmol^-1^ and established four H-bonding interactions with DNA-polymerase molecule indicating its strong inhibitory effect (Fig. 5). One H-bond was established between the hydrogen atom of a hydroxyl group of L6 and the carbonyl oxygen of residue GLN17; the hydrogen atom of another hydroxyl group of L6 was involved in the second H-bond with the carbonyl oxygen of residue ILE216. The third H-bond was developed between the hydrogen atom of the third hydroxyl group of L6 and the carbonyl oxygen of residue LYS238; the fourth H-bond was formed between the oxygen atom of the same third hydroxyl group of L6 and the α-hydrogen atom of residue LYS238 (Table S3). L5 showed three physical interactions with the receptor 4TR6 molecule resulting in binding energy of -8.6359 Kcalmol^-1^ (Fig. S4).

**Table 7:** Docking results of tentatively identified compounds (L1-L9) against receptor 4TR6

Receptors	Ligands	Number of interactions	Binding energy (Kcalmol^-1^)	Interacting residues
4TR6	L1 L2 L3 L4 L5 L6 L7 L8 L9	02 01 02 05 03 04 05 03 03	-6.2235 -5.7973 -7.3578 -5.5456 -8.6359 -9.9476 -8.4604 -8.2522 -7.0680	LYS190, GLN260 ASP18 LEU217, ASP218 ILE216, ASP18, LYS238, ASP218, ASP18 LYS 238, LYS 238, LEU223 ILE216, LYS238, GLN17, LYS238 2ASP18, LYS21, LEU217, ASP218 LEU217, LYS21, ASP218 ILE216, LYS238, ILE216

Secondary metabolites L7, L8, and L9 displayed 5, 3, and 3 physical interactions with receptor 4TR6 molecule, respectively with their binding energies of -8.4604, -8.2522, and -7.0680 Kcalmol^-1^, respectively (Fig. S5). These results infer that these ligands also have strong potential for the inhibition of the DNA-polymerase enzyme. Compounds L1, L2, L3, and L4 exhibited 2, 1, 2, and 5 interactions with DNA-polymerase molecules, respectively (Fig. S6). These physical interactions resulted binding energies of -6.2235, -5.7973, -7.3578, and -5.5456 Kcal mol^-1^, respectively [26, 27].

**Fig. 6:**
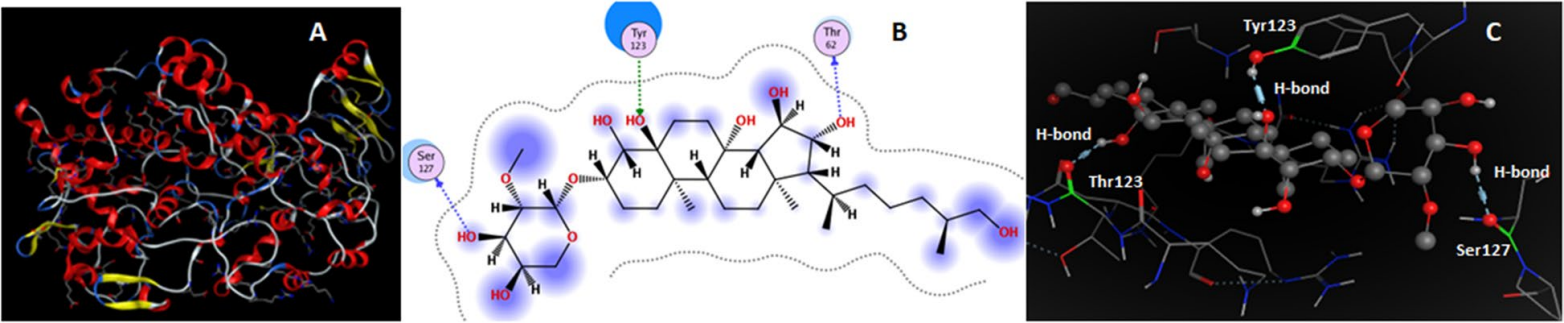
(**A**) 3D structure of COX-2, (**B**) 2D and (**C**) 3D interactions of **L6** with COX-2

Similarly, compound L6 generated three H-bonding interactions with the COX-2 enzyme resulting the highest binding affinity of -9.9476 Kcal mol^-1^ among all docked compounds (Fig. 6). One H-bond was developed between the hydrogen atom of a hydroxyl group of L6 and the carbonyl oxygen of SER127; Second H-bond was established between the oxygen atom of a hydroxyl group of L6 and the hydrogen atom of the hydroxyl group of residues TYR123. Carbonyl oxygen of residue THR62 and hydrogen atom of a hydroxyl group of L6 were involved in the third H-bond formation (Table S4). Strong physical interactions and binding affinity make L6 a potential candidate for the inhibition of the COX-2 enzyme. Compounds L7, L8, and L9 have high binding energies of -8.4604, -8.2522, and -7.0680 Kcalmol^-1^ as well as 4, 2, 1 physical interaction values with COX-2, respectively (Fig. S7). Binding energies of L7, L8, and L9 indicate their high inhibitory effects. Similarly, mycocompounds L1-L5 possessed reasonable physical interactions (Fig. S8) and binding affinities with COX-2 protein (Table 8).

**Table 8:** Docking results of tentatively identified compounds (L1-L9) against receptor 5JVZ

Receptor	Ligands	Number of interactions	Binding energy (Kcal mol^**-1**^)	Interacting residues
5JVZ	L1 L2 L3 L4 L5 L6 L7 L8 L9	01 01 03 07 03 03 04 02 01	-6.2235 -5.7973 -8.0984 -5.5456 -8.6359 -9.9476 -8.4604 -8.2522 -7.0680	TYR123 SER127 LYS79, ASP126, SER127 CYS41, GLU466, ASN43, 2LYS469, ARG44, GLU466 PHE372, ASP126, SER127 THR62, SER127, TYR123 TYR123, LYS469, SER472, ARG44 SER472, ASP126 TYR123

Docking results deduce that mycocompound L6 has the strongest potential for the inhibition of both DNA-polymerase of *Bacillus subtilis* and inflammation supporting enzyme cyclooxygenase-2. Other compounds also carry reasonable inhibitory effects [26, 27, 60]. Keeping in view their strong physical interactions and binding affinities, tentatively identified compounds support the antibacterial and anti-inflammatory effects of *A. ficuum*.

## Conclusions

The drug discovery procesas been advanced by exploring fungi for their distinctive pharmacological and other beneficial aspects. In this study, we examined the metabolic profile of *A. ficuum* for the first time using GC-MS and LC-QToF-MS techniques. The metabolic profile indicates the presence of several compounds of pharmacological and biological importance. Similarly, we screened *A. ficuum* pharmacologically in an *in vivo* and *in vitro* model for the first time. Preliminary results revealed that both ethyl acetate and n-hexane fractions significantly inhibit the growth of standard bacterial pathogens. *A. ficuum* extract also possessed the highest DPPH radicals scavenging effect. Ethyl acetate extract displayed the highest anti-inflammatory effect on paw edema in mice, which was further confirmed by observing an increase in the percentage inhibition of inflammation. Complimentary molecular docking analysis of tentatively identified compounds with the DNA polymerase enzyme of *Bacillus subtilis* and the pro-inflammatory enzyme COX-2 also supported the antibacterial and anti-inflammatory activities of the *A. ficuum* extract. Ligand L6 showed strong interactions with both target proteins and could therefore be used as a potent inhibitor against the various pathogenic enzymes. In addition, no acute toxicity was reported for *A. ficuum*. These results suggest that *Aspergillus* has the potential to be used as a promising therapeutic for drug discovery in the future.

## Supplementary Information

**Additional file 1: Supplementary Figure S1**. Structures of docked ligands (L1-L9). **Figure S2.** Percentage antioxidant activity of n-hexane and ethyl acetate fractions. **Figure S3.** (A) Oral administration of AF extract, (B) Injection of carrageenan in paw, (C**,** D, E) measurement of Paw edema at regular intervals. **Figure S4.** (**A**) 2D and (**B**) 3D interactions of L5 with DNA-polymerase enzymes of *bacillus subtilis.* **Figure S5.** (**A, C, E**) 2D and (**B, D, F**) 3D interactions of L7, L8 and L9 with DNA-polymerase enzymes of *bacillus subtilis*, respectively. **Figure S6.** (**A, C, E, G**) 2D and (**B, D, F, H**) 3D interactions of L1, L2, L3 and L4 with DNA-polymerase enzymes of *bacillus subtilis*, respectively. **Figure S7.** (**A, C, E**) 2D and (**B, D, F**) 3D interactions of L7, L8, L9 and COX-2, respectively. **Figure S8. A, C, E, G**) 2D and (**B, D, F, H**) 3D interactions of L1, L2, L3, L4 and L5 with COX-2, respectively.

**Additional file 2: Table S1**. Complete detail of LC-MS-QTOF analysis of *Aspergillus ficuum*.

**Additional file 3: Table S2**. Complete detail of GC-MS analysis of *Aspergillus ficuum*.

**Additional file 4: Table S3**. Nature, distance and energy of interactions of secondary metabolites (L1-L9) with receptor 4TR6.

**Additional file 5: Table S4**. Nature, distance and energy of interactions of secondary metabolites (L1-L9) with receptor 5JVZ.

## Data Availability

The datasets generated and/or analyzed during this study are available from the corresponding author upon reasonable request.
