# Supplementary material for: Insights into metabolic and pharmacological profiling of Aspergillus ficuum through bioinformatics and experimental techniques
Source: BMC Microbiol. 2022 Dec 9;22:295. doi: 10.1186/s12866-022-02693-w (PMC9733250; doi:10.1186/s12866-022-02693-w)
Supplement: Supplementary file 1 — Additional file 1: Supplementary Figure S1. Structures of docked ligands (L1-L9). Figure S2. Percentage antioxidant activity of n-hexane and ethyl acetate fractions. Figure S3. (A) Oral administration of AF extract, (B) Injection of carrageenan in paw, (C, D, E) measurement of Paw edema at regular intervals. Figure S4. (A) 2D and (B) 3D interactions of L5 with DNA-polymerase enzymes of bacillus subtilis. Figure S5. (A, C, E) 2D and (B, D, F) 3D interactions of L7, L8 and L9 with DNA-polymerase enzymes of bacillus subtilis, respectively. Figure S6. (A, C, E, G) 2D and (B, D, F, H) 3D interactions of L1, L2, L3 and L4 with DNA-polymerase enzymes of bacillus subtilis, respectively. Figure S7. (A, C, E) 2D and (B, D, F) 3D interactions of L7, L8, L9 and COX-2, respectively. Figure S8. A, C, E, G) 2D and (B, D, F, H) 3D interactions of L1, L2, L3, L4 and L5 with COX-2, respectively. [file 12866_2022_2693_MOESM1_ESM.docx]

Supplementary Materials

**Insight into Metabolic and Pharmacological Profiling of *Aspergillus ficuum* through Bioinformatics and Experimental Techniques**

**Zafar Ali Shah^1,2^, Khalid Khan^1*^, Haroon Ur Rashid^3^, Tanzeel Shah^4^, Mariusz Jaremko^5*^, Zafar Iqbal^2^**

^1*^Department of Chemistry, Islamia College, Peshawar, KP, Pakistan

^2^Department of Agricultural Chemistry & Biochemistry, University of Agriculture, Peshawar, KP, Pakistan

^3^Department of Chemistry, Federal University of Santa Catarina, Florianopolis, SC, Brazil.

^4^Institute of Basic Medical Sciences, Khyber Medical University, Peshawar, KP, Pakistan

^5^**^*^**[Biological and Environmental Science and Engineering Division](https://bese.kaust.edu.sa/), King Abdullah University of Science and Technology, [Thuwal](https://en.wikipedia.org/wiki/Thuwal), [Makkah Province](https://en.wikipedia.org/wiki/Makkah_Province), 23955-6900, [Saudi Arabia](https://en.wikipedia.org/wiki/Saudi_Arabia)

*Corresponding authors E-mail addresses: [drkhalidchem@yahoo.com](mailto:drkhalidchem@yahoo.com), [Mariusz.jaremko@kaust.edu.sa](mailto:Mariusz.jaremko@kaust.edu.sa)

Table of contents

1. **Fig. S1** Structures of docked ligands (L1-L9).
2. **Fig. S2** Percentage antioxidant activity of n-hexane and ethyl acetate fractions.
3. **Fig. S3** (A) Oral administration of AF extract, (B) Injection of carrageenan in paw, (C**,** D, E) measurement of Paw edema at regular intervals.
4. **Fig. S4** (**A**) 2D and (**B**) 3D interactions of L5 with DNA-polymerase enzymes of *bacillus subtilis.*
5. **Fig. S5** (**A, C, E**) 2D and (**B, D, F**) 3D interactions of L7, L8 and L9 with DNA-polymerase enzymes of *bacillus subtilis*, respectively.
6. **Fig. S6** (**A, C, E, G**) 2D and (**B, D, F, H**) 3D interactions of L1, L2, L3 and L4 with DNA-polymerase enzymes of *bacillus subtilis*, respectively.
7. **Fig. S7** (**A, C, E**) 2D and (**B, D, F**) 3D interactions of L7, L8, L9 and COX-2, respectively.
8. **Fig. S8 A, C, E, G**) 2D and (**B, D, F, H**) 3D interactions of L1, L2, L3, L4 and L5 with COX-2, respectively.
9. **Table S1** Complete detail of LC-MS-QTOF analysis of *Aspergillus ficuum*
10. **Table S2** Complete detail of GC-MS analysis of *Aspergillus ficuum*
11. **Table S3** Nature, distance and energy of interactions of secondary metabolites (L1-L9) with receptor 4TR6.
12. **Table S4** Nature, distance and energy of interactions of secondary metabolites (L1-L9) with receptor 5JVZ.


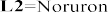


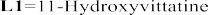


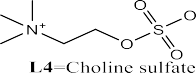

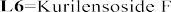

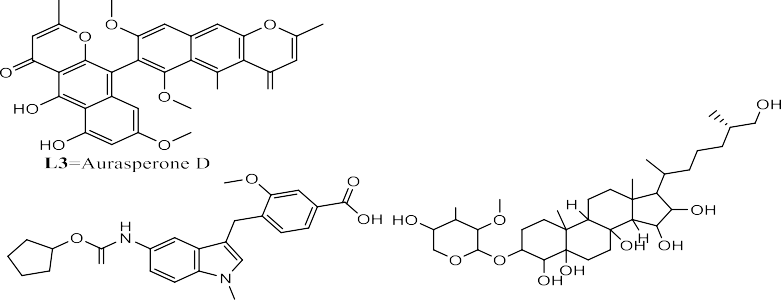


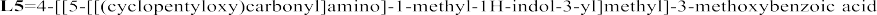


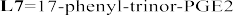


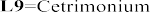


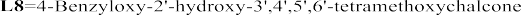
**Fig. S1** Structures of docked ligands (L1-L9).


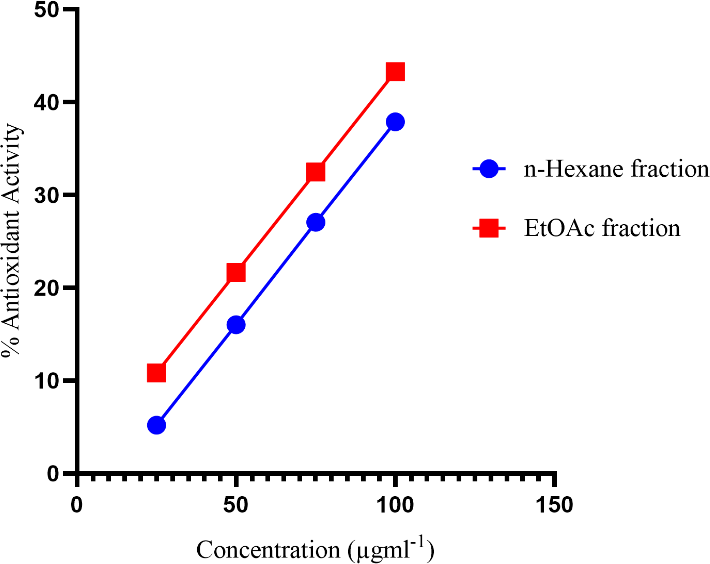


**Fig. S2** Percentage antioxidant activity of n-hexane and ethyl acetate fraction**s.**


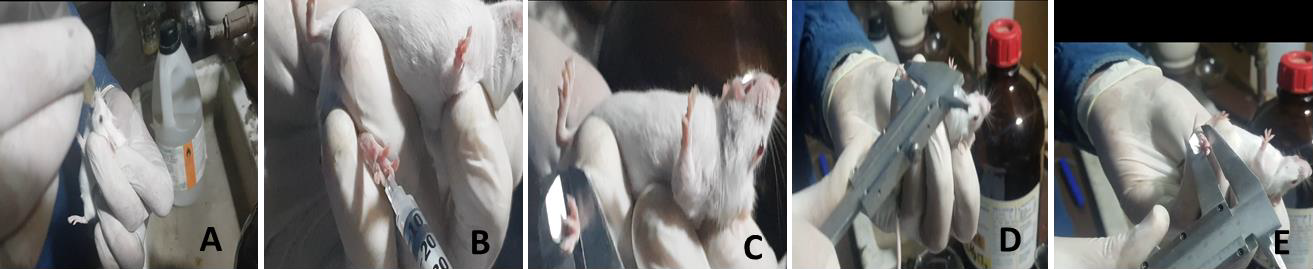


**Fig. S3** (A) Oral administration of AF extract, (B) Injection of carrageenan in paw, (C**,** D, E) measurement of Paw edema at regular intervals.


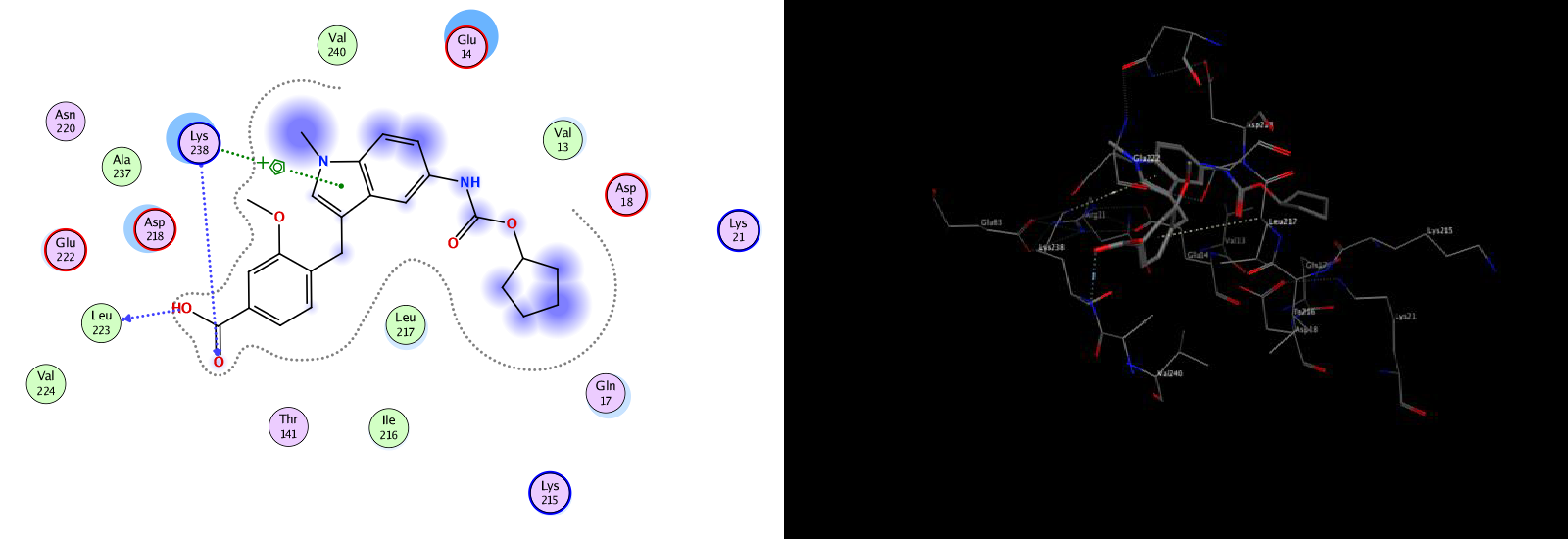


**A**

**B**

**Fig. S4** (**A**) 2D and (**B**) 3D interactions of L5 with DNA-polymerase enzymes of *bacillus subtilis.*


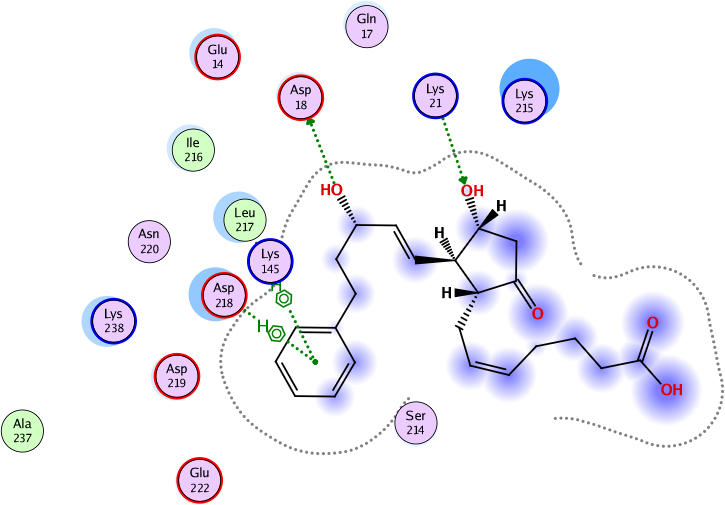


**A**


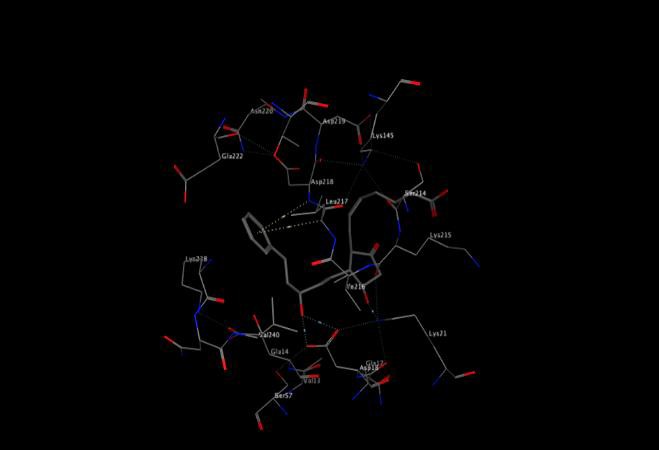


**B**


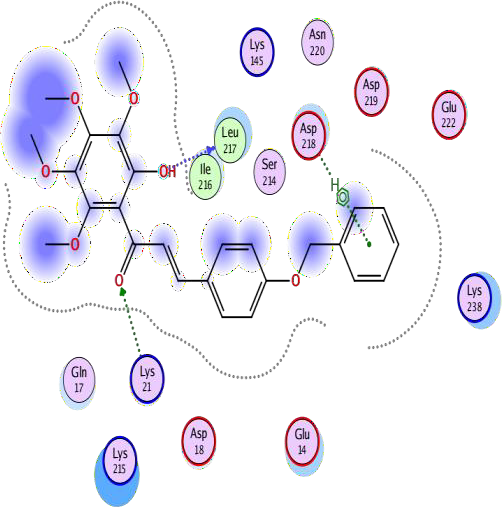


**C**


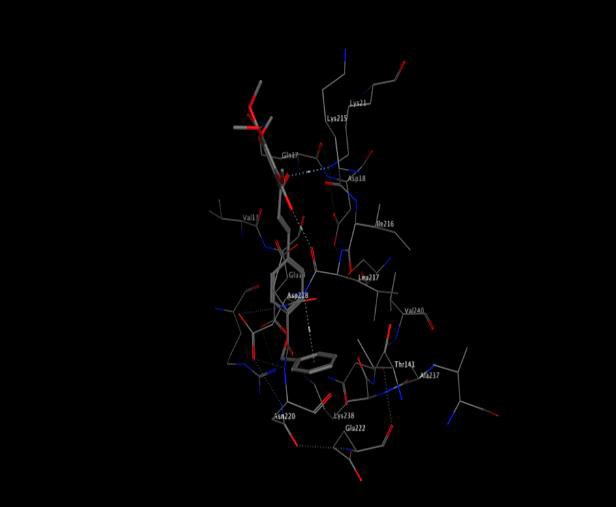


**D**


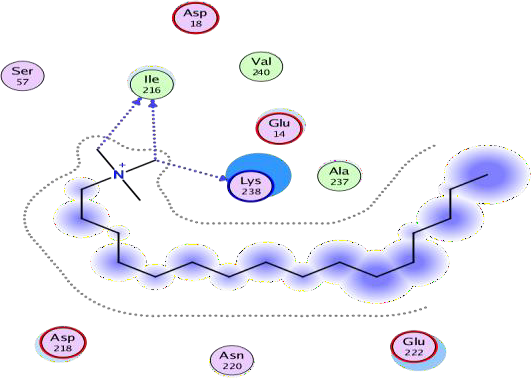


**E**


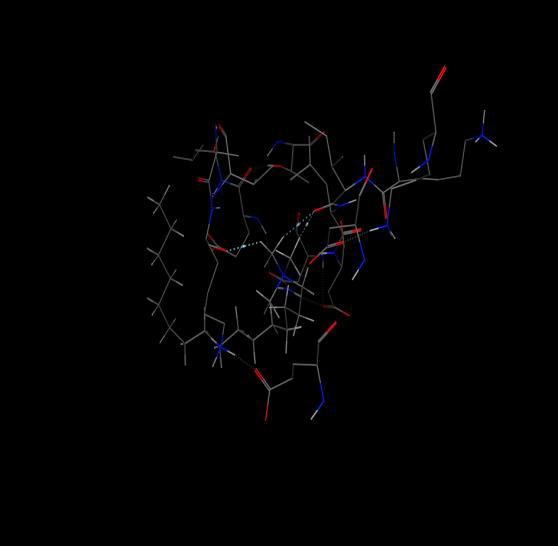


**F**

**Fig. S5** (**A, C, E**) 2D and (**B, D, F**) 3D interactions of L7, L8 and L9 with DNA-polymerase enzymes of *bacillus subtilis*, respectively.


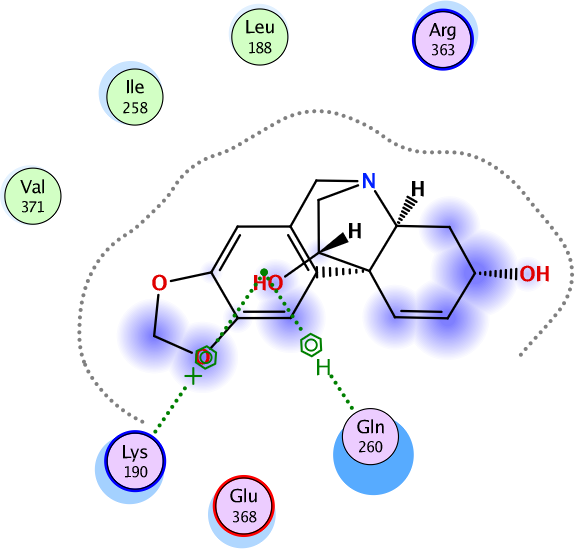


**A**


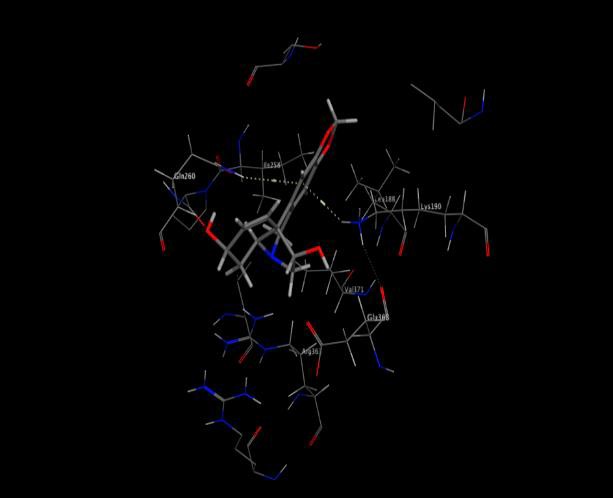


B


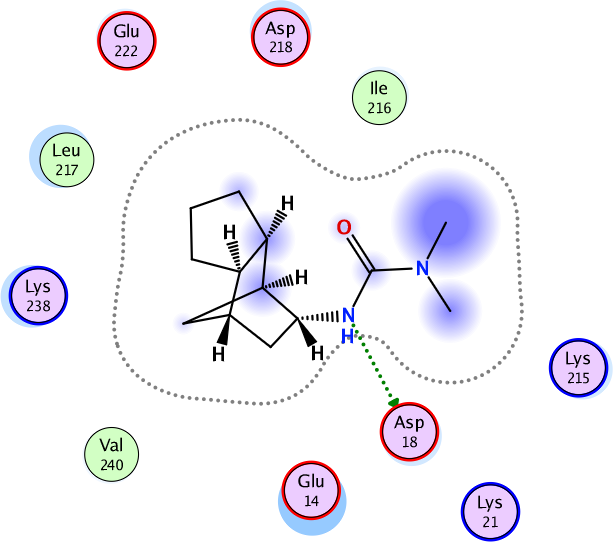


**C**


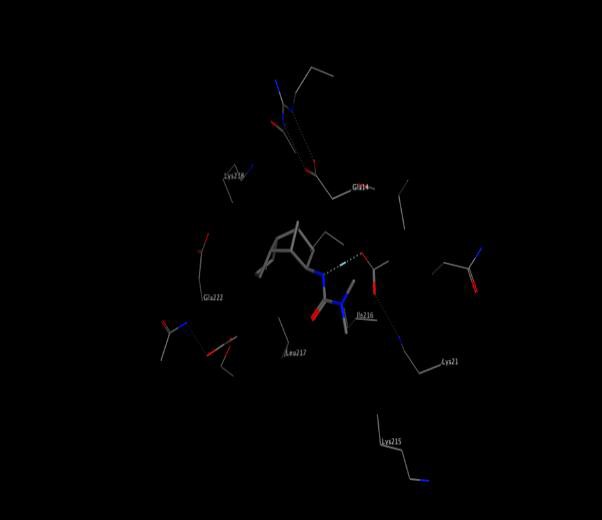


**D**


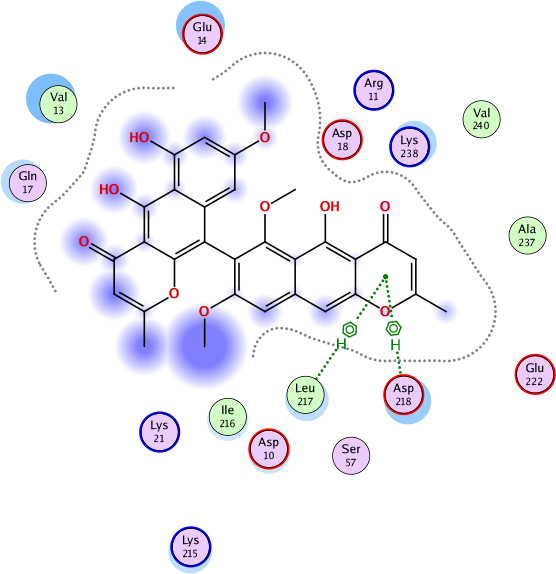


**E**


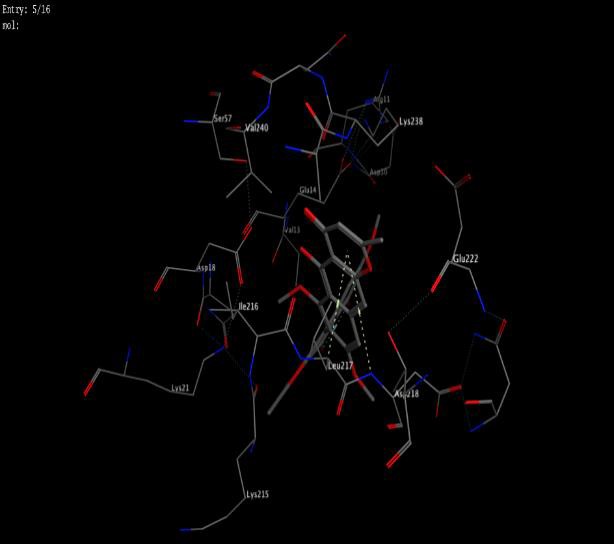


**F**


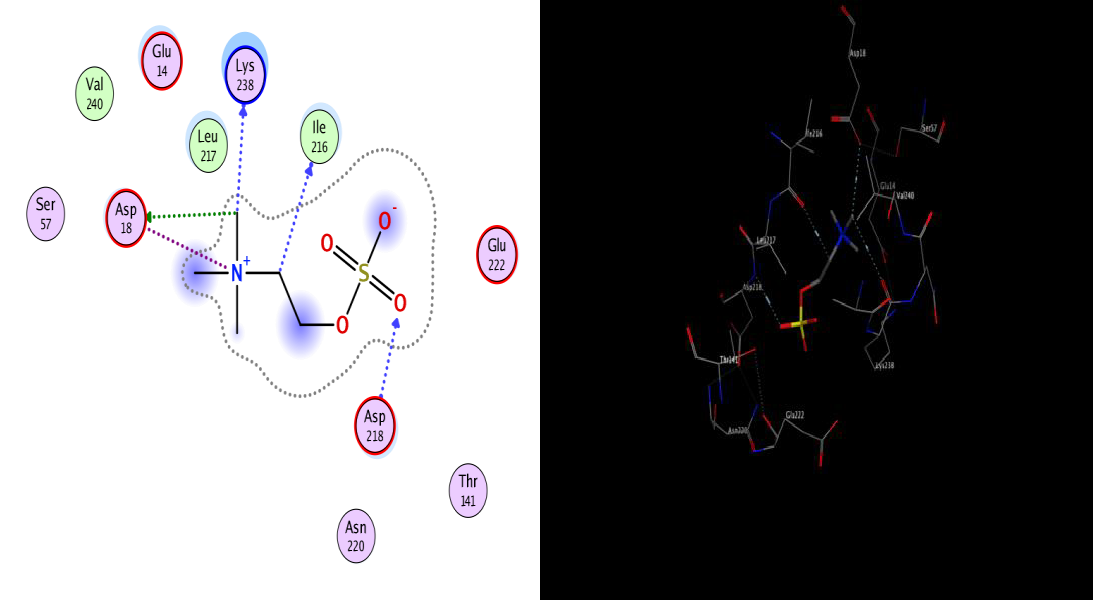


**G**

**H**

**Fig. S6** (**A, C, E, G**) 2D and (**B, D, F, H**) 3D interactions of L1, L2, L3 and L4 with DNA-polymerase enzymes of *bacillus subtilis*, respectively.


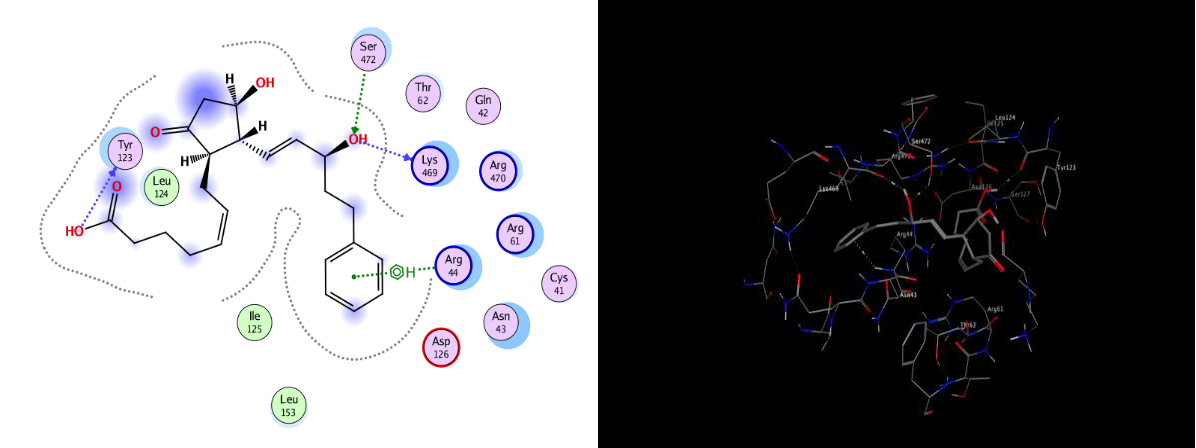


**A**

**B**


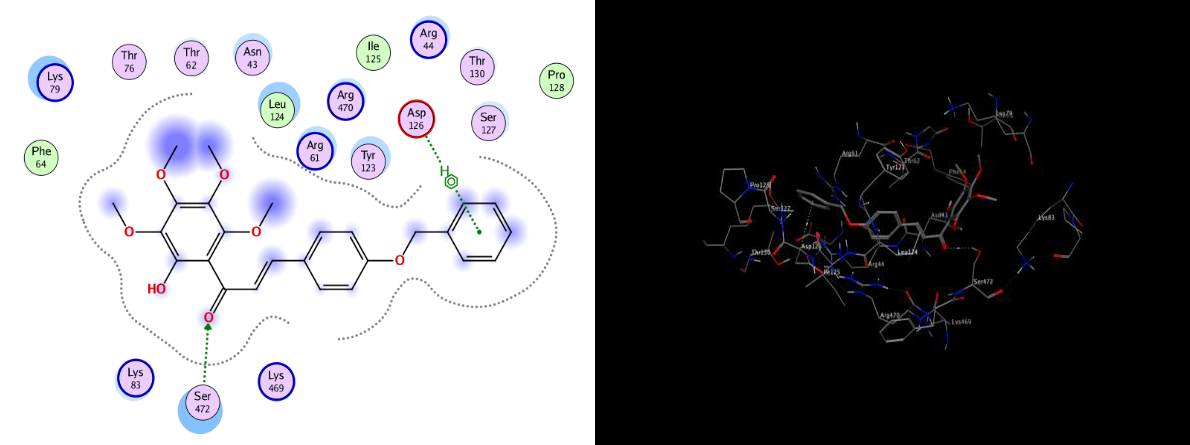


**C**

**D**


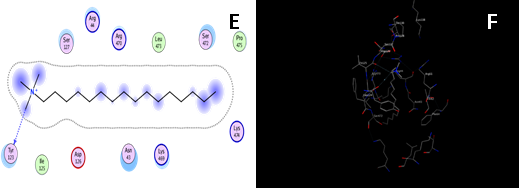


**Fig. S7** (**A, C, E**) 2D and (**B, D, F**) 3D interactions of L7, L8, L9 and COX-2, respectively.


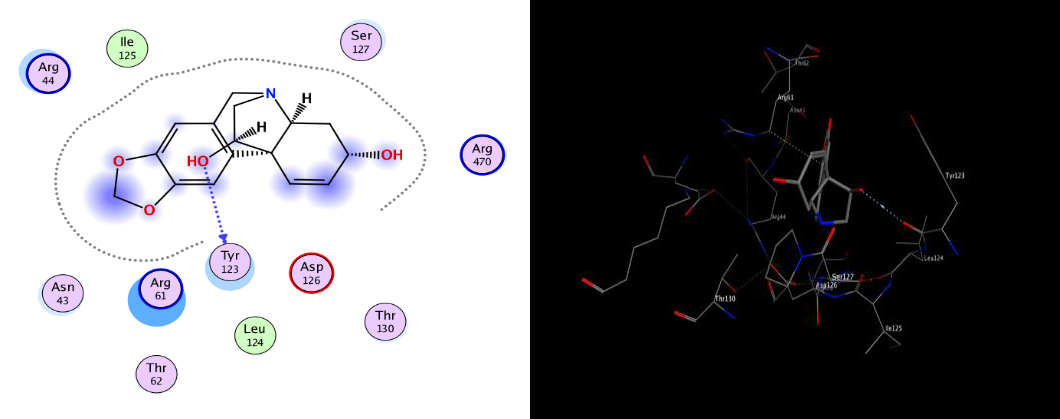


**A**

**B**


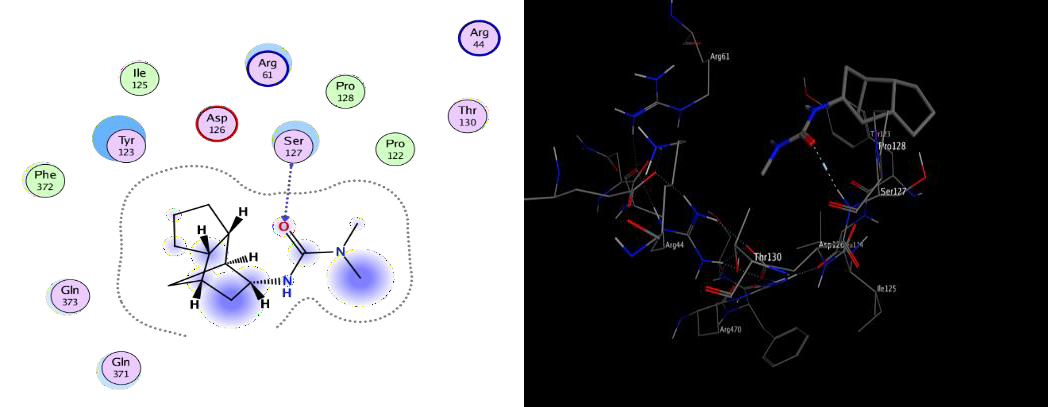


**C**

**D**


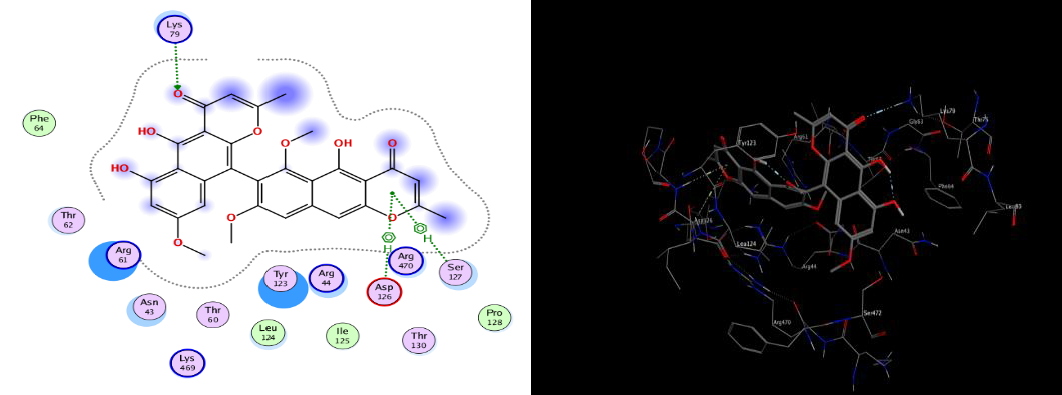


**E**

**F**


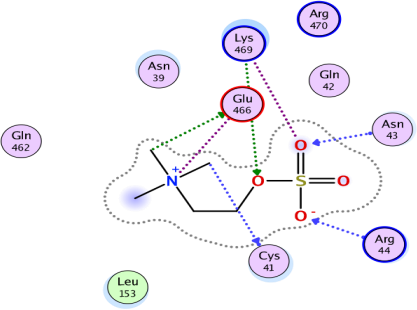


**G**


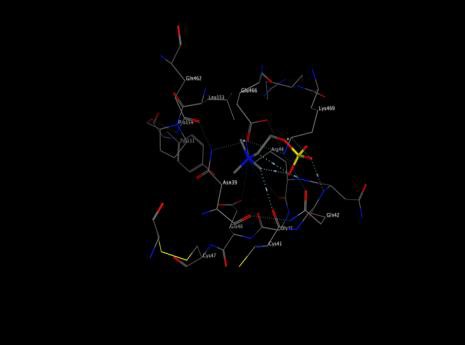


**H**


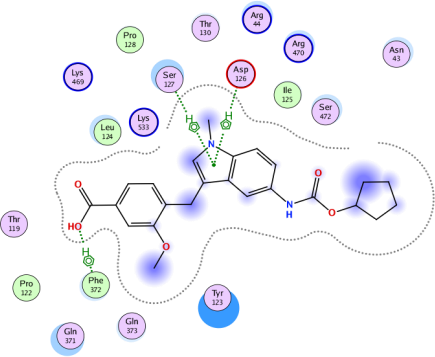


**I**


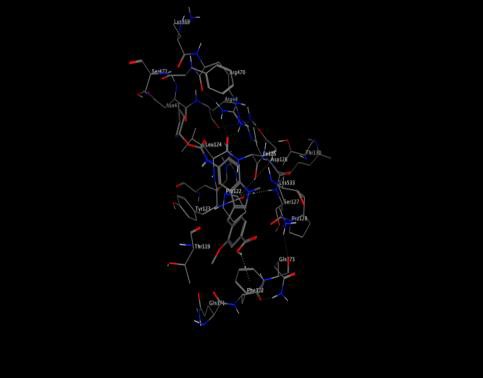


**J**

**Fig. S8 A, C, E, G**) 2D and (**B, D, F, H**) 3D interactions of L1, L2, L3, L4 and L5 with COX-2, respectively.

**Table S1** Original file provided in MS Excel as a separate supplementary material

**Table S2** Original file provided in MS Excel as a separate supplementary material

**Table S3** Nature, distance and energy of interactions of secondary metabolites (L1-L9) with receptor 4TR6.

| **Ligand** | **Nature of interaction** | **Distance (A0)** | **E (Kcalmol-1)** |
| --- | --- | --- | --- |
| L1 | pi-cation | 3.56 | -1.9 |
|  | pi-H | 4.53 | -0.6 |
| L2 | H-donor | 2.97 | -3.3 |
| L3 | pi-H | 4.18 | -1.6 |
|  | pi-H | 4.11 | -1.0 |
| L4 | H-donor | 3.15 | -1.6 |
|  | H-donor | 3.31 | -0.8 |
|  | H-donor | 3.52 | -0.8 |
|  | H-acceptor | 3.15 | -3.4 |
|  | Ionic | 3.98 | -0.5 |
| L5 | H-donor | 2.85 | -1.0 |
|  | H-acceptor | 2.63 | -1.1 |
|  | pi-cation | 4.33 | -1.0 |
| L6 | H-donor | 2.74 | -1.9 |
|  | H-donor | 2.55 | -1.2 |
|  | H-donor | 2.90 | -1.7 |
|  | H-acceptor | 2.95 | -1.1 |
| L7 | H-donor | 3.28 | -0.6 |
|  | H-donor | 2.97 | -1.1 |
|  | H-acceptor | 3.01 | -1.0 |
|  | pi-H | 4.30 | -1.1 |
|  | pi-H | 4.15 | -0.7 |
| L8 | H-donor | 2.89 | -1.0 |
|  | H-acceptor | 2.94 | -1.5 |
|  | pi-H | 4.41 | -1.8 |
| L9 | H-donor | 3.30 | -0.9 |
|  | H-donor | 3.15 | -0.9 |
|  | H-donor | 3.21 | -1.1 |

**Table S4** Nature, distance and energy of interactions of secondary metabolites (L1-L9) with receptor 5JVZ.

| **Ligand** | **Nature of interaction** | **Distance (A0)** | **E (Kcalmol-1)** |
| --- | --- | --- | --- |
| L1 | H-donor | 3.11 | -1.4 |
| L2 | H-acceptor | 3.14 | -1.9 |
| L3 | H-acceptor | 3.38 | -3.3 |
|  | pi-H | 4.42 | -1.6 |
|  | pi-H | 4.04 | -3.0 |
| L4 | H-donor | 3.12 | -1.4 |
|  | H-donor | 3.21 | -0.9 |
|  | H-acceptor | 2.99 | -0.9 |
|  | H-acceptor | 3.06 | -1.0 |
|  | H-acceptor | 2.73 | -5.1 |
|  | ionic | 3.92 | -0.7 |
|  | Ionic | 3.95 | -0.6 |
| L5 | H-pi | 5.56 | -0.6 |
|  | pi-H | 4.57 | -0.7 |
|  | pi-H | 4.13 | -1.0 |
| L6 | H-donor | 3.15 | -1.3 |
|  | H-donor | 3.19 | -1.3 |
|  | H-acceptor | 2.65 | -1.5 |
| L7 | H-donor | 3.05 | -0.7 |
|  | H-donor | 2.91 | -2.1 |
|  | H-acceptor | 3.12 | -0.6 |
|  | pi-H | 3.40 | -1.0 |
| L8 | H-acceptor | 3.10 | -0.9 |
|  | pi-H | 3.98 | -0.8 |
| L9 | H-donor | 3.35 | -0.8 |
