# Supplementary material for: Insights into metabolic and pharmacological profiling of Aspergillus ficuum through bioinformatics and experimental techniques
Source: BMC Microbiol. 2022 Dec 9;22:295. doi: 10.1186/s12866-022-02693-w (PMC9733250; doi:10.1186/s12866-022-02693-w)
Supplement: Supplementary file 4 — Additional file 4: Table S3. Nature, distance and energy of interactions of secondary metabolites (L1-L9) with receptor 4TR6. [file 12866_2022_2693_MOESM4_ESM.docx]

**Table S3** Nature, distance and energy of interactions of secondary metabolites (L1-L9) with receptor 4TR6

| **Ligand** | **Nature of interaction** | **Distance (A0)** | **E (Kcalmol-1)** |
| --- | --- | --- | --- |
| L1 | pi-cation | 3.56 | -1.9 |
|  | pi-H | 4.53 | -0.6 |
| L2 | H-donor | 2.97 | -3.3 |
| L3 | pi-H | 4.18 | -1.6 |
|  | pi-H | 4.11 | -1.0 |
| L4 | H-donor | 3.15 | -1.6 |
|  | H-donor | 3.31 | -0.8 |
|  | H-donor | 3.52 | -0.8 |
|  | H-acceptor | 3.15 | -3.4 |
|  | Ionic | 3.98 | -0.5 |
| L5 | H-donor | 2.85 | -1.0 |
|  | H-acceptor | 2.63 | -1.1 |
|  | pi-cation | 4.33 | -1.0 |
| L6 | H-donor | 2.74 | -1.9 |
|  | H-donor | 2.55 | -1.2 |
|  | H-donor | 2.90 | -1.7 |
|  | H-acceptor | 2.95 | -1.1 |
| L7 | H-donor | 3.28 | -0.6 |
|  | H-donor | 2.97 | -1.1 |
|  | H-acceptor | 3.01 | -1.0 |
|  | pi-H | 4.30 | -1.1 |
|  | pi-H | 4.15 | -0.7 |
| L8 | H-donor | 2.89 | -1.0 |
|  | H-acceptor | 2.94 | -1.5 |
|  | pi-H | 4.41 | -1.8 |
| L9 | H-donor | 3.30 | -0.9 |
|  | H-donor | 3.15 | -0.9 |
|  | H-donor | 3.21 | -1.1 |
