# Supplementary material for: Insights into metabolic and pharmacological profiling of Aspergillus ficuum through bioinformatics and experimental techniques
Source: BMC Microbiol. 2022 Dec 9;22:295. doi: 10.1186/s12866-022-02693-w (PMC9733250; doi:10.1186/s12866-022-02693-w)
Supplement: Supplementary file 5 — Additional file 5: Table S4. Nature, distance and energy of interactions of secondary metabolites (L1-L9) with receptor 5JVZ. [file 12866_2022_2693_MOESM5_ESM.docx]

**Table S4** Nature, distance and energy of interactions of secondary metabolites (L1-L9) with receptor 5JVZ

| **Ligand** | **Nature of interaction** | **Distance (A0)** | **E (Kcalmol-1)** |
| --- | --- | --- | --- |
| L1 | H-donor | 3.11 | -1.4 |
| L2 | H-acceptor | 3.14 | -1.9 |
| L3 | H-acceptor | 3.38 | -3.3 |
|  | pi-H | 4.42 | -1.6 |
|  | pi-H | 4.04 | -3.0 |
| L4 | H-donor | 3.12 | -1.4 |
|  | H-donor | 3.21 | -0.9 |
|  | H-acceptor | 2.99 | -0.9 |
|  | H-acceptor | 3.06 | -1.0 |
|  | H-acceptor | 2.73 | -5.1 |
|  | ionic | 3.92 | -0.7 |
|  | Ionic | 3.95 | -0.6 |
| L5 | H-pi | 5.56 | -0.6 |
|  | pi-H | 4.57 | -0.7 |
|  | pi-H | 4.13 | -1.0 |
| L6 | H-donor | 3.15 | -1.3 |
|  | H-donor | 3.19 | -1.3 |
|  | H-acceptor | 2.65 | -1.5 |
| L7 | H-donor | 3.05 | -0.7 |
|  | H-donor | 2.91 | -2.1 |
|  | H-acceptor | 3.12 | -0.6 |
|  | pi-H | 3.40 | -1.0 |
| L8 | H-acceptor | 3.10 | -0.9 |
|  | pi-H | 3.98 | -0.8 |
| L9 | H-donor | 3.35 | -0.8 |
